# The architectural history of the “new buildings” of the Medical University of Vienna

**DOI:** 10.1007/s00508-025-02619-2

**Published:** 2025-09-11

**Authors:** Markus Müller

**Affiliations:** Medical University of Vienna, Vienna, Austria

**Keywords:** Vienna Medical School, Vienna General Hospital, AKH, Eric Kandel, Precision medicine, Translational medicine

## Abstract

The current buildings of the Medical University of Vienna were mainly constructed between the eighteenth and twentieth centuries and reflect a multigenerational ambition to keeping up with the latest developments in medicine. The current comprehensive structural expansion of the Medical University of Vienna within about a decade represents a significant turning point. Following the construction of the old and the new Vienna General Hospital (AKH), the establishment of modern infrastructure by the preclinical “MedUni Campus Mariannengasse” (MCM), the Center for Translational Medicine (CTM), and Center for Precision Medicine (CPM)/“Eric Kandel Institute” represents another milestone for Austrian medicine and will accompany the next generations of Austrian physicians for many decades.

## Introduction

“Build it and they will come” and “In America, university presidents are judged by the number of cranes they set up” are two remarks from a member of the Scientific Advisory Board of the Medical University of Vienna that underscore the special importance of construction projects for a university’s attractiveness and development within the framework of international competition. The current buildings of the Medical University of Vienna, which were constructed between the eighteenth and twentieth centuries represent historical documents of this multigenerational ambition. At the same time, they reflect medical progress, which was manifested in several phases. In the construction of the “old” Vienna General Hospital (AKH), at a time of extremely high infant mortality, low life expectancy and lack of suitable diagnostic and therapeutic procedures, the primary focus was on welfare and the idea of a care facility for the poor and destitute (“pre-scientific phase”). The enlightened, science-based approach of the First and Second Vienna Medical School [1, 2] resulted in completely new requirements for medical care, teaching, and research buildings. The construction of the “new” Vienna General Hospital (AKH) was a landmark project for Austrian Medicine, representing a classical example of the era of “science-based repair medicine”. At the beginning of the twenty-first century, artificial intelligence, translational medicine and precision medicine are once again creating new structural challenges in a phase of “science-based preventive medicine”. Due to the ongoing comprehensive structural expansion and renovation of the Medical University of Vienna (https://bauprojekte.meduniwien.ac.at/), the current history of these developments is described in more detail.

## Short history of the infrastructure of the Medical University of Vienna (1784–1964)

**Fig. 1:**
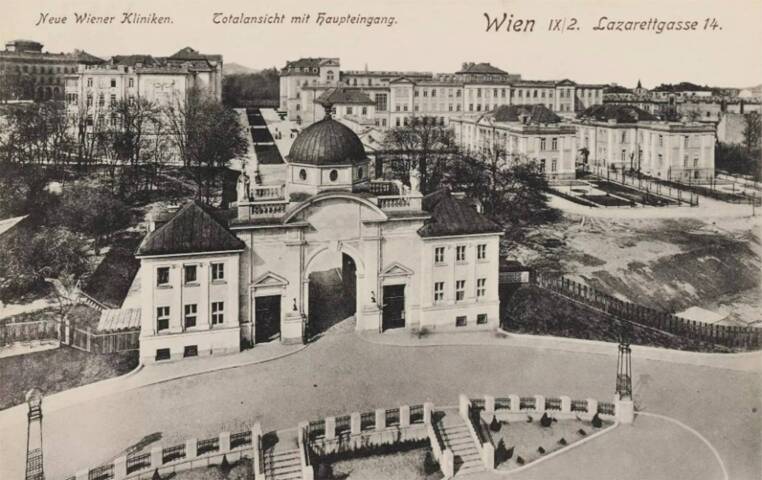
1914: new departments of the “old” Vienna General Hospital (AKH) Wien, view from main entrance 9., Lazarettgasse 14. Ansichtskarte, 1913–1914 (Herstellung), Paul Ledermann (Hersteller), Wien Museum Inv.-Nr. 58891/885, CC0 (https://sammlung.wienmuseum.at/objekt/117971/) open content

During the First Vienna Medical School, founded by Gerard van Swieten, the “Great Poorhouse” in Alsergrund was converted into the General Hospital (“AKH”) in 1782 and was opened as the University Hospital by Joseph II on August 16, 1784. A second architectural milestone followed in 1785 with the opening of the Josephinum, a military surgical academy ([3], https://www.josephinum.ac.at/). In the mid-nineteenth century, the Faculty of Medicine was expanded with buildings for preclinical institutes on the site of a former imperial and royal rifle factory near Währinger Straße. In 1904, at the height of the Second Vienna Medical School, Franz Joseph I laid the cornerstone for an AKH extension with “new clinics” in the area of Spitalgasse on the Alserbach Hill, where the two Women’s Clinics (“Wertheim”/“Schauta”), the 1st Medical Clinic (“Wenkebach”/“Deutsch”), the ENT Clinic, the Children’s Clinic (“Pirquet”) and the Neurological Clinic (“Wagner-Jauregg”) were built ([4]; Fig. 1).

**Fig. 2:**
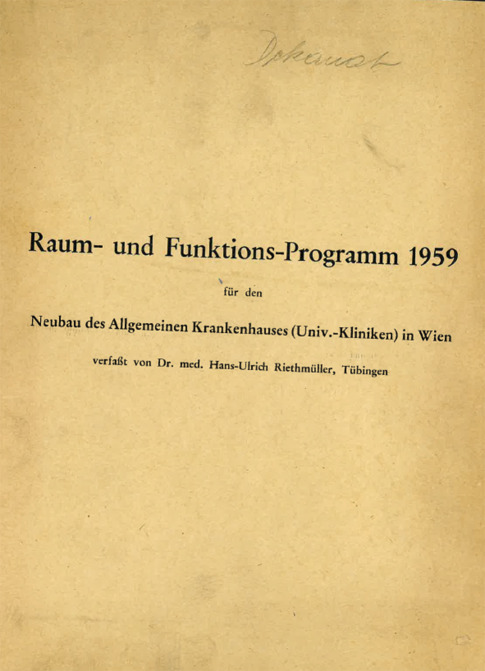
1959: Front page for the spatial and functional concept of the “new” Vienna General Hospital (AKH); archive of MedUni Wien

The University Hospital AKH remained under the auspices of the Imperial and Royal (“k.&k.”) Administration until 1918 and became a federal hospital of the Republic of Austria during the First Republic. In 1939, during the Nazi era, the AKH became property of the city of Vienna. As noted in a parliamentary report, “after the restoration of democratic conditions in Austria, a fragmentation of legal ownership took place between the federal government, the City of Vienna, and the University sector … The return of responsibilities to one hand, as had been the case before 1938, did not occur” [5]. This type of management and the distribution of responsibilities between the federal government, the City of Vienna and the University were recurring themes in the post-war period [5, 6]. In addition, the increasingly desolate structural situation and deplorable working conditions at the old AKH after 1945 became an obstacle to meeting international standards of academic medicine [6]. As early as 1955 the first concepts for a new AKH building were developed under Prof. Karl Fellinger who later became Rector of the University. These plans conceived a location at the outskirts of Vienna at “Steinhof”, including a “multiple pavillion complex” for the University Hospital and also the preclinical departments [6]. In 1959, however, a spatial and functional concept for a single, central building in the city center on the Alserbach hill was developed ([7, 8]; Fig. 2) and the first construction phase began in 1964 [8].

## Construction and operation of the “new” general hospital “AKH” (1964–2016)

In 1984, the clinics at the “Südgarten” (pediatrics, psychiatry, neurosurgery) were put into operation. In 1994, after successive settlement, the “new” Vienna General Hospital was officially opened as one of the largest hospitals in Europe at the time. These construction projects were accompanied by corruption trials and parliamentary investigative committees [5, 6, 9]. Reports to the investigative committee and parliamentary statements of facts noted, among other things, that “actually no one had a clear overview” [5]. Despite all the difficulties, the Vienna General Hospital became a success story and is now, once again, ranked among the best university hospitals in the world (https://rankings.newsweek.com/worlds-best-hospitals-2025). As early as the 1990s, various plans for the construction of patient hotels or garages by investors were discussed. The University unsuccessfully pursued the concept of an “integrated campus” by rebuilding the outdated preclinical institutes on the Vienna General Hospital site.

The separation of the Medical Faculty from the University of Vienna in 2004, creating a Medical University independent of the Federal Government, marked a turning point in the organizational structure that had been repeatedly considered unsatisfactory. As a result of the separation, the “old” Vienna General Hospital clinics, with few exceptions such as the current Brain Research Center, became institute buildings of the University of Vienna and still house numerous humanities institutes.

**Fig. 3:**
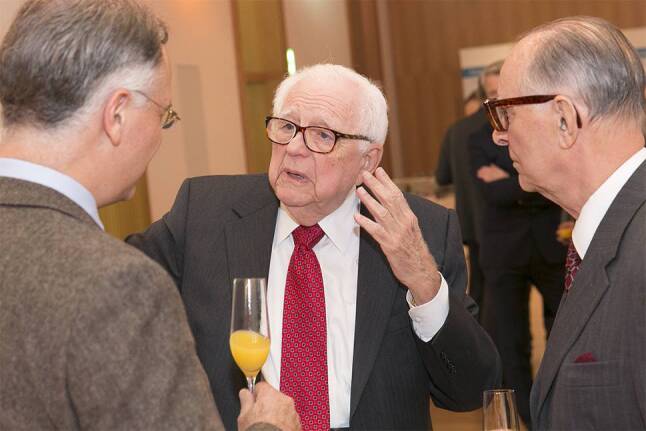
2014: Prof. Eugene Braunwald (middle), Prof. Oleh Hornykiewicz (right) and Prof. Markus Müller (left) discussing the importance of infrastructure and translational Medicine; archive of MedUni Wien 2014

The rapid progress in the field of molecular precision medicine led to new university buildings worldwide, especially in Asia and the USA. In 2015, under the leadership of Vice Rector-designate Oswald Wagner and Rector-designate Markus Müller, a conceptual plan “Future Development of the AKH Medical University Campus” was presented. This plan was also based on discussions following a lecture by Prof. Eugene Braunwald of Harvard University on the occasion of the University’s 10th anniversary (Fig. 3). This plan envisioned structural measures for innovative medicine, such as the construction of a Center for Translational Medicine (CTM), a Center for Precision Medicine (CPM) for the use of high-end molecular biology for modern diagnostic and therapeutic procedures, and a Technology Transfer Center (TTC) for the economic utilization of research results and for intensified collaboration with industrial partners. At the same time, the City of Vienna and private health insurers developed a plan to build a private hospital on the grounds of the Vienna General Hospital (AKH), in exchange for land owned by the “Confraternität/Goldenes Kreuz” private hospital. This plan was discontinued following a meeting between Mayor Michael Häupl and Rector Markus Müller.

## New buildings at the “MedUni Campus AKH” (2016–2027)

In 2013, a Federal Court of Audit report (“Federal-State Cooperation in the Health Care System Using the Vienna General Hospital as an Example”) heavily criticized the cooperation between the Federal Government and the City of Vienna (https://www.parlament.gv.at/dokument/XXIV/III/437/imfname_320365.pdf). The General Hospital was reported as “expensive, inefficient, and bureaucratic,” and “the auditors gave the major hospital a devastating economic and administrative assessment” [10]. After intense negotiations about new terms of cooperation, three new agreements were signed by the Federal Government, the City, and the University on 27 January 2016: 1) a “cooperation agreement” between the University and the City of Vienna for a joint AKH management, 2) a “target management agreement” for regulating investment and operating cost flows, and 3) a “framework construction agreement” (RBV) for AKH building reinvestments of approximately € 1.3 billion until 2030. Within the framework of the RBV, the Medical University of Vienna was contractually granted “irrevocable, perpetual, unrestricted, and free-of-charge rights of use” of the AKH facilities and equipment. During these negotiations on the RBV, the University concept developed in 2015 was also accepted as the basis for planning the CTM and CPM.

The entire architectural planning followed the concept of a “productivity chain” for the benefit of patients, the scientific community and technology transfer: 1) routine care using established procedures at the AKH, 2) experimental care using innovative procedures at the CTM, 3) basic research focused on molecular precision medicine at the CPM, 4) commercial exploitation of biomedical innovations together with industry at the TTC. The kick-off of links 2) and 3) will take place successively over the course of 2026, only the planning of the TTC is currently not completed.

### Center for Precision Medicine (CPM)/Eric Kandel Institute:

The planning work for the CPM, with approximately 6500 m^2^ of usable space (13,000 m^2^ gross space), went hand in hand with the CTM planning. This planning was made possible by an intensive fundraising campaign for the CPM starting in 2016. On 14 February 2020, a demolition notice for the old buildings (“Klinik Deutsch”) on the construction site was officially granted. These preparations formed the basis of an application to the European Union’s European Resilience and Recovery Facility (EFFR), announced by Rector Markus Müller and Chancellor Sebastian Kurz on 26 April 2021. The Facility submitted a full commitment to CPM funding on 21 June 2021. In 2021, Prof. Eric Kandel permitted the CPM to be named the “*Eric Kandel Institute*” due to his personal relationship to the Medical University. The CPM will create modern conditions for research on personalized and digital diagnostics and therapeutics. The CPM will also temporarily house the “*Ignaz Semmelweis Institute*”, operated jointly by all Austrian Medical Universities, for a couple of years. It began operations on 1 January 2025. The CPM foundation stone was laid on 15 December 2023, in the presence of Professor Eric Kandel and Mayor Michael Ludwig. The topping-out ceremony took place on September 17, 2025. The building will be gradually opened in 2026 (https://bauprojekte.meduniwien.ac.at/).

### Center for Translational Medicine (CTM) und Braunwald Lecture Hall:

**Fig. 4:**
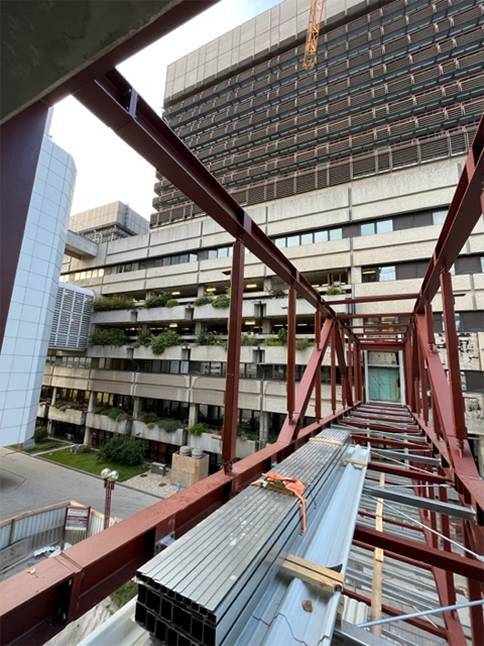
2024: Construction of the connecting bridge between the main Vienna General Hospital (AKH) complex and the Center for Translational Medicine, a symbol for medical and architectural translation

The slogan “from bench to bedside and back again” for the CTM, which is funded by the Federal Government and the City through the RBV, underscores the idea of making the benefits of basic research available to patients as quickly as possible while simultaneously leveraging experience from patient care for research projects. The CTM is thus conceived as a hub for innovative patient care. A phase I/II center, core facilities, a GMP facility for cell therapy, radiopharmacy, and biologicals, modern imaging, bioinformatics, and a biobank will be located on approximately 14,000 m^2^ of usable floor space. The CTM, with a new University auditorium with approximately 800 seats, named the “*Eugene Braunwald Lecture Hall*” in honor of Professor Braunwald, is directly connected to the CPM and the AKH core building via bridges (Fig. 4). Together with the new preclinical campus “MedUni Campus Mariannengasse” (MCM), which is within sight of the new campus, it forms the center of an integrated, completely new healthcare, research and teaching architecture. The topping-out ceremony for the CTM took place on 17 October 2024, and the opening will take place gradually in 2026 (https://bauprojekte.meduniwien.ac.at/).

### Anna Spiegel II:

Starting in 2022, the existing Anna Spiegel research building was expanded by approximately 4700 m^2^ gross space as part of the RBV. Starting in 2025, research departments from various University hospitals will relocate to the new Anna Spiegel II building, where modern research spaces have been established, also connected to the CTM and CPM. The space thus freed up in the main building will be used to expand patient care areas (https://bauprojekte.meduniwien.ac.at/). The west façade of the building is adorned with an artificial intelligence (AI) reconstruction of Gustav Klimt’s famous faculty painting “Medicine” [11].

## A new building for the preclinical institutes: MedUni Campus Mariannengasse “MCM” (2015–2027)

Partly as a result of the historic defeat in the Battle of Königgrätz in 1866, parts of an Imperial and Royal (“k.&k.”) rifle factory, which had supplied the Austrian army for many decades, were converted into preclinical institutes of the Faculty of Medicine between 1854 and 1886. This complex, housing institutes for anatomy, histology, physiology, and pharmacology, served many generations of Austrian physicians for over 150 years as a training facility and also as an object of emotional identification, but has required multiple renovations over the past several decades. The plan for an “integrated campus” by rebuilding the outdated preclinical institutes on the AKH site was already pursued in the 1990s but was never realized. An opportunity to integrate the MedUni Campus arose in 2012 under the University’s founding rector, Wolfgang Schütz, when the University acquired the “Wien Energie Building” in the Mariannengasse/Spitalgasse area. After the property was sold to the Federal Real Estate Company (BIG) for University use and used as an asylum center during the 2015 European refugee crisis, the construction of the new campus was agreed upon on 16 December 2016, following negotiations between the University and the Federal Ministry of Education, Science, Research and Economy (BMBWF) as part of the 2016–2018 performance agreement. An architectural competition was held in 2018, the official building permit was issued on 18 August 2022, and in 2023, partial demolition of the partially listed building began, and the foundation stone for the major MCM construction project was laid. At the MCM, the previously scattered preclinical facilities will be consolidated on a usable area of approximately 35,000 m^2^ (60,000 m^2^ gross space), creating modern facilities for teaching and training. The three large lecture halls will be located on the ground floor of the main building, and a cafeteria will be located on the ground floor of the listed existing building. A direct public passageway to the MedUni Campus AKH will be created across the MCM in the direction of Lazarettgasse. Starting in 2027, approximately 2000 students will attend courses at the MCM, and approximately 800 employees from the Centers for Physiology and Pharmacology, Anatomy and Cell Biology, Pathobiochemistry and Genetics, Medical Physics and Biomedical Engineering, and the Center for Cancer Research will relocate to the MCM (https://bauprojekte.meduniwien.ac.at/).

## Josephinum, Himberg and new monuments

The Josephinum [3] is undoubtedly one of the world’s most important museums of medical history and, at the same time, the most beautiful building of the Medical University. Designed by Isidor Canevale (1730–1786), the building was opened in 1785 and houses a unique collection of anatomical wax figures from Florence, comprising 1200 models. In 2018, the Josephinum had to be closed due to a lack of building technology and air conditioning, which endangered the valuable collections. Thanks to a budget agreed upon by the University, the BIG (Federal Real Estate Company), and the responsible Vice Chancellor Reinhold Mitterlehner, the building was completely renovated and reopened on 29 September 2022, after almost 4 years of renovation. A special feature of the renovation is the restoration of the historic lecture hall, in which remnants of the original eighteenth century wall paintings were rediscovered and restored.

In 2021, a new building for laboratory animal breeding and husbandry, including a hybrid operating room, laboratory, training, and office space, was finalized in Himberg, which also serves as a testing and training center for new medical devices.

A special feature of the recent construction work is a historic Sigmund Freud monument by the Jewish sculptor Oscar Nemon, donated in 2018 by the Freud and Nemon families (https://www.meduniwien.ac.at/web/ueber-uns/news/detailseite/2018/news-im-juni-2018/zu-ehren-sigmund-freuds-statue-an-der-meduni-wien-enthuellt/) as well as an Ignaz Semmelweis monument, unveiled in 2019 as a gift from the Hungarian state (https://www.meduniwien.ac.at/web/ueber-uns/news/detailseite/2019/news-im-februar-2019/statue-von-ignaz-semmelweis-an-der-meduni-wien-enthuellt/).

**Fig. 5:**
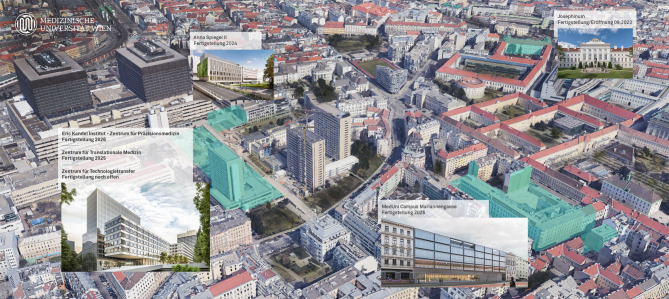
Overview map of the buildings at the MedUni Campus Vienna General Hospital (AKH) (Centers for Translational Medicine, Precision Medicine, Anna Spiegel II), at the MedUni Campus Mariannengasse and at the Josephinum; archive of MedUni Wien 2025

The comprehensive structural expansions and renovations of the Medical University of Vienna within about a decade (Fig. 5), thus represent a turning point in the history of Viennese Medicine [1, 2, 12–14] following the construction of the old and new Vienna General Hospital and the acquisition of the preclinical buildings in the nineteenth century. The modern infrastructure of the MCM, CTM, and CPM will certainly accompany the next generations of Austrian physicians for many decades in the current phase of preventive medicine, fuelled by artificial intelligence and molecular precision methods.
